# Q-slope and SSAM applied to excavated coal mine slopes

**DOI:** 10.1016/j.mex.2020.101191

**Published:** 2020-12-28

**Authors:** Neil Bar, Alison McQuillan

**Affiliations:** Gecko Geotechnics, Cairns, Australia

**Keywords:** Slope stability, Q-slope, Open cut coal

## Abstract

The Q-slope classification system is used to assess the stability of excavated rock slopes and provide an indication of long-term stable, reinforcement-free slope angles. Q-slope is based on over 500 rock slope case studies from mines, road and rail cuttings hosted in igneous, sedimentary and metamorphic rocks around the world. Q-slope can be applied for slopes ranging from less than 5 m to more than 250 m in height in both civil and mining environments.

This paper describes the application of Q-slope classification system to 38 failed and intact slopes from Australian open cut coal mines. It further describes the relationship between Q-slope and Slope Stability Assessment Methodology (SSAM) ratings for stable slopes based on the available case studies.

## Graphical abstract

**Figure:**
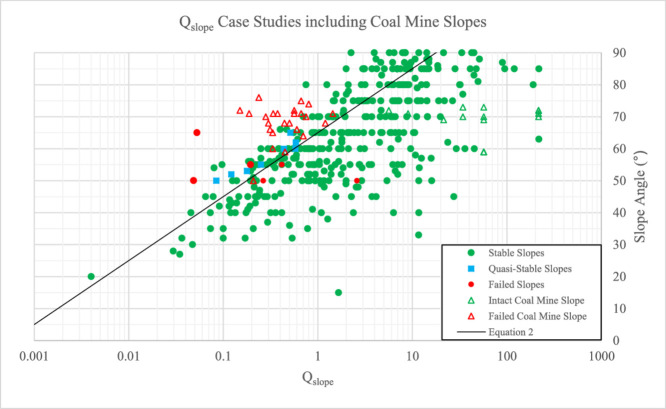

Specification table

**Table:** 

Subject Area	Engineering
More specific subject area	Geotechnical Engineering Rock Mechanics Slope Stability
Method name	Q-slope, SSAM
Name and reference of original method	Barton, N. and Bar, N. (2015). Introducing the Q-slope method and its intended use within civil and mining engineering projects. Future Development of Rock Mechanics; Proc. ISRM Regional Symposium Eurock 2015 & 64th Geomechanics Colloquium, Salzburg, Austria, 157–162. McQuillan, A., Canbulat, I., Payne, D. and Oh, J. (2018). New risk assessment methodology for coal mine excavated slopes. International Journal of Mining Sciences and Technology, 28(4), 583–592. DOI: https://doi.org/10.1016/j.ijmst.2018.07.001
Resource availability	Q-slope: https://play.google.com/store/apps/details?id=com.geckogeotech.q_slope&hl=en SSAM: https://app.ssam.net.au/

## *Method details

In this study, 38 failed and stable slope cases from Australian open cut coal mines have been assessed using the Q-slope classification system. A relationship is given for stable, quasi-stable and failed slopes based on Q-slope rating. Further, a relationship is defined between Q-slope and SSAM (Slope Stability Assessment Methodology), two empirical classification systems for predicting slope stability.

Q-slope ratings are calculated using Eq. (1) [1].

(1) $$Q_{\text{slope}}\;=\;\frac{\text{RQD}}{J_{n}}\;\times \;{\left(\frac{J_{r}}{J_{a}}\right)}_{0}\;\times \;\frac{J_{\text{wice}}}{\text{SR}F_{\text{slope}}}$$

where RQD = rock quality designation [4]; Jn = joint set number; Jr = joint roughness number; Ja = joint alteration number; Jwice = environmental and geological condition number, and SRFslope = the maximum strength reduction factor for weathering, low strength and/or faulted zones that may adversely affect slope stability.

SSAM ratings are determined by selecting the slope conditions most applicable from the list of critical parameters defined in Table 1 [3].

**Table 1:** The SSAM classification technique for excavated coal mine slopes. Ratings assigned to each critical parameter are highlighted in Italics to the left of each Slope Condition description.

Critical Parameter		Slope Condition							
**1**	**Rock Mass Unit**	*1*	Massive: No persistent beds	*5*	Interbedded – Fine: 2+ persistent beds with average bedding thickness < 5 m	*10*	Interbedded – Coarse: 2+ persistent beds with average bedding thickness 5–10 m	*15*	Massive: 2+ persistent beds with average bedding thickness > 10 m
**2**	**Structure – orientation relative to excavated hardwall**	*1*	No persistent structure OR 1+ persistent discontinuity striking > 30° from hardwall orientation	*15*	2+ intersecting persistent discontinuities, with 1 persistent discontinuity set striking 〈 50° and 1+ persistent discontinuity set striking 〉 50° relative to the excavated hardwall orientation			*30*	1+ persistent discontinuity striking < 30° from hardwall orientation OR 2+ intersecting persistent discontinuities both striking < 50° relative to the excavated hardwall orientation
**3a**	**Structure dip** **1 persistent discontinuity**	*1*	Structure dip < 80° into the face OR no persistent discontinuities	*5*	Structure dip < 40° into the excavation	*15*	Structure dip > 60° into the excavation	*20*	Structure dip 40 to 60° into the excavation OR structure dip 80 to 90° into the face
**3b**	**Structure dip** **2+ persistent discontinuities**			*5*	Structure dip < 40° into the excavation	*15*	Structure dip 40 to 60° into the excavation	*20*	Structure dip > 60° into the excavation
**4**	**Lateral conditions**	*1*	Strata/bedding is horizontal or dips away from the face	*10*	Strata/bedding locally rolls or dips into the face			*20*	Strata/bedding consistently rolls or dips into the face AND/OR a coal or carbonaceous band is present at crest or base of persistent structure
**5**	**Water**	*1*	No water seepage OR Dry slope conditions	*10*	Consistent water seepage out of face (i.e. stable head)			*20*	Change in seepage conditions (e.g. sudden new, increase, decrease, or stoppage in seepage conditions without causal weather event OR water ponding at crest OR saturated at toe
**6**	**Wall geometry**	*1*	Straight, no inflections OR elbows	*10*	Concave inflection/*s* < 180 °	*15*	Convex inflection/*s* > 180 °	*20*	90° elbow (bullnose)
**7**	**Weathering**	*1*	Fresh: no orange staining on defect surfaces OR in fresh horizon	*10*	Moderately weathered: some orange staining on defect surfaces – may be in weathered or fresh horizon			*20*	Extremely weathered: > 70% orange staining on defect surfaces OR in weathered horizon
**8**	**Structure surface waviness**	*1*	Wavy, several undulations	*5*	Wavy, moderate undulations	*10*	Smooth, low undulations OR known previous shearing on discontinuity surface OR surface conditions unknown		
**9**	**Height**	*1*	> 20 m	*5*	21 to 40 m	*10*	41 to 60 m	*15*	> 60 m
**10**	**Angle**	*1*	< 62 °	*5*	63 to 67 °	*10*	68 to 72 °	*15*	> 73 °

**Fig. 1:**
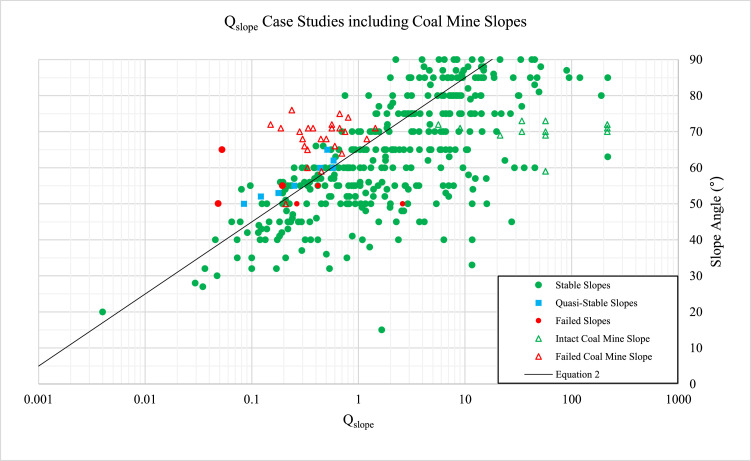
Q-slope ratings and slope angles for over 500 cases studies including coal mine slopes.

Fig. 1 displays slope performance and Q-slope rating for 531 slope cases (384 intact, 8 quasi-stable and 139 failed cases).

On the applicability of Q-slope to predict excavated coal mine slope performance,  Fig. 1 illustrates a good correlation between Q-slope's prediction of stability, Eq. (2) [2].

(2) $$\beta \;=\;20\;\text{lo}g_{10}Q_{\text{slope}}+\;65^{\circ }$$

where *β* = the steepest slope angle not requiring reinforcement or support.

**Fig. 2:**
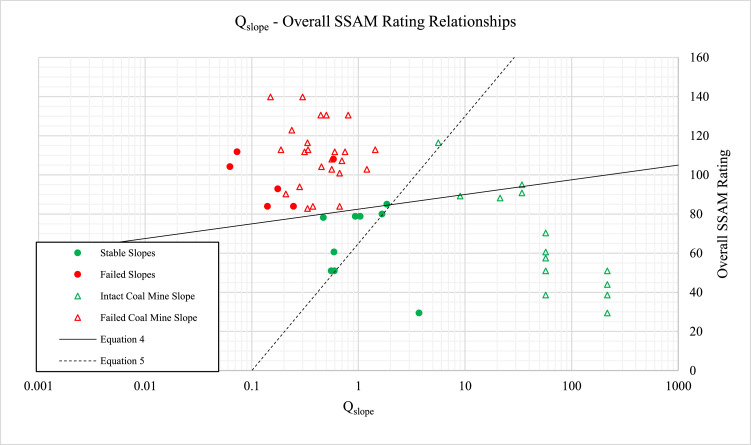
Relationship between Q-slope and the overall SSAM rating for 54 slope cases.

Q-slope and SSAM ratings, Eq. (3), calculated for the same open cut coal mine slopes, were compared to determine any correlation between Q-slope and SSAM, Fig. 2.

(3) $$\text{LOF}\;=\;\frac{1}{1\;+\;e^{(6.860\;-\;\left(0.0769\;\times \;\text{overall}\;\text{SSAM}\;\text{rating}\right))}}$$

where SSAM = overall SSAM rating as calculated from conditions selected in Table 1.

Fig. 2 indicates two correlations between Q-slope and the overall SSAM rating for stable slopes. Eq. (4) is a line-of-best fit indicating the main boundary between stable and failed slopes. For Q-slope values equal to, or exceeding 2, the upper bound Eq. (5), may also be used to estimate SSAM. However, Eq. (5) should be cautiously used given it is based on relatively limited data.

(4) $$\text{Overall}\;\text{SSAM}\;\text{Rating}\;=\;7.5\text{lo}g_{10}Q_{\text{slope}}+\;82.5$$

(5) $$\text{Upper}\;\text{Bound}\;\text{Overall}\;\text{SSAM}\;\text{Rating}\left(\text{when}\;Q_{\text{slope}}\geq 2\right)=\;65\;\text{lo}g_{10}Q_{\text{slope}}+\;65$$

**Fig. 3:**
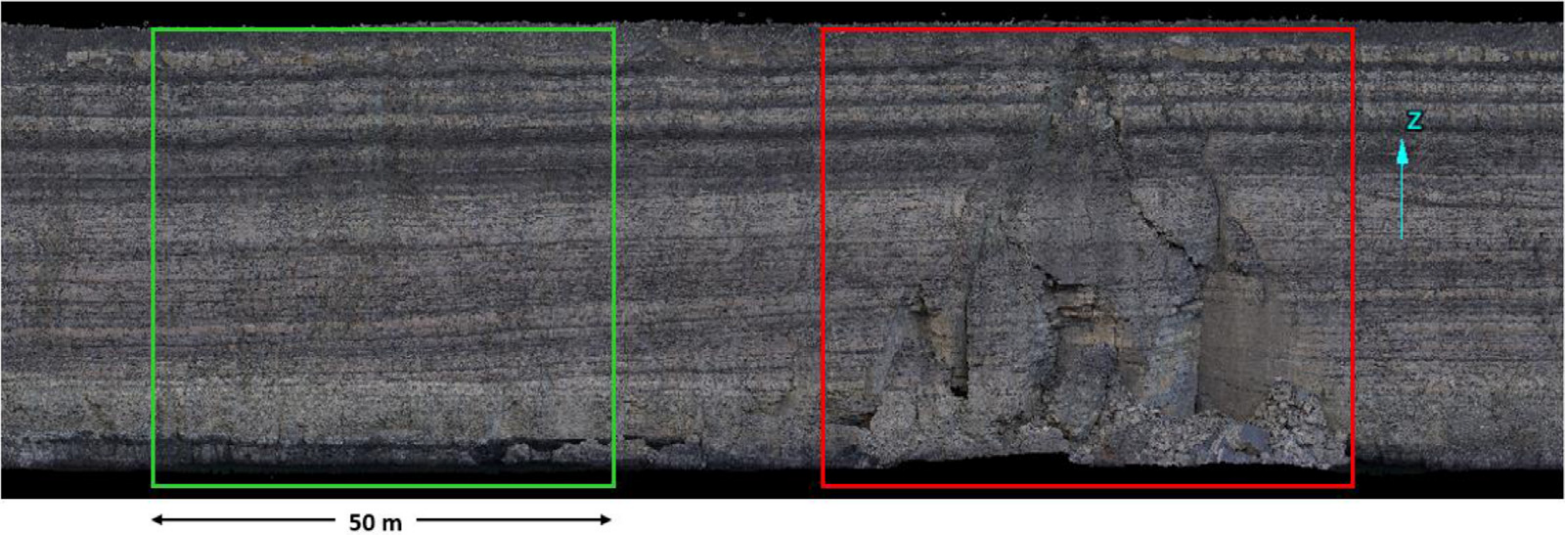
Case study – an intact section of slope is highlighted by the green polygon and a failed section of slope is highlighted by the red polygon.

An example application of Q-slope and SSAM to an excavated coal mine slope is also included. The example case study slope was 46 m high and had an as-built slope geometry of 66° This case study, shown in Fig. 3, represents a single geotechnical domain (i.e. zone of expected similar ground behaviour), exhibiting both an intact section of slope (highlighted in the green) and a failed section of slope (highlighted by the red). Both highlighted sections are bounded by two intersecting sub-vertical discontinuities projected to form a wedge [3].

An overall SSAM rating of 112 was estimated, which corresponds to a LOF of 85% as per a summation of the following conditions:

1. 10: Interbedded – Coarse: 2+ persistent beds with average thickness 5–10 m
2. 30: 2+ intersecting persistent discontinuities both striking < 50° relative to the excavated hardwall orientation
3. 15: Structure dip 40–60° into the excavation (3b).
4. 20: Strata consistently rolls or dips into the face
5. 1: No water seepage or dry slope conditions
6. 1: Straight wall geometry, no inflections or elbows
7. 10: Moderately weathered
8. 10: Smooth, low undulations
9. 10: Batter height of 41 to 60 m
10. 5: Slope angle of 63 to 67°

A Q-slope value of 1.0 was estimated for the case study slope, per the following inputs:

• Average RQD = 90% across varying interbedded strata of varying rock mass quality. Note: RQD was estimated visually from a distance of approximately 15 metres from the actual slope.
• J_n_ = 9
• Set A: J_r_ = 1, J_a1_ = 1, O_factor_ = 0.5
• Set B: J_r_ = 1, J_a1_ = 1, O_factor_ = 0.8
• J_wice_ = 1
• SRF_slope_ = 4.

Q-slope is estimated to be 1.0 equates to a stable slope angle, β, of 65° (i.e. slightly shallower than the actual slope angle).

## Declaration of Competing Interest

The authors declare that they have no known competing financial interests or personal relationships that could have appeared to influence the work reported in this paper.
